# Surgery for bronchiectasis: The effect of morphological types to prognosis

**DOI:** 10.4103/1817-1737.74273

**Published:** 2011

**Authors:** Ufuk Cobanoglu, Irfan Yalcinkaya, Metin Er, Ahmet Feridun Isik, Fuat Sayir, Duygu Mergan

**Affiliations:** *Department of Thoracic Surgery, University of Yuzuncu Yil, Van, Turkey*; 1 *Department of Sureyyapasa Chest Diseases and Surgery Education and Research Hospital, Istanbul, Turkey*; 2 *Dr. Suat Seren Chest Diseases and Surgery Education and Research Hospital, Izmir, Turkey*; 3 *University of Gaziantep, Gaziantep, Turkey*

**Keywords:** Bronchiectasis, prognosis, saccular type, surgical treatment, tubular-type

## Abstract

**BACKGROUND:**

Although the incidence has declined over the past years in societies with high socioeconomic status, bronchiectasis is still an important health problem in our country.

**AIM:**

To review and present our cases undergoing surgery for bronchiectasis in the past 12 years and their early and late term postoperative outcomes and our experience in bronchiectasis surgery and the effect of morphological type on the prognosis.

**METHODS:**

The medical records of 62 cases undergoing surgical resection for bronchiectasis in the Clinics of Thoracic and Pediatric Surgery were evaluated retrospectively. The disease was on the left in 33 cases, on the right in 26 and bilateral in three cases. The most common surgical procedure was lobectomy. Forty one patients underwent pneumonectomy, lobectomy and complete resection including bilobectomy. Twenty-one (33.87%) cases underwent incomplete resection, of whom 11 (17.74%) underwent segmentectomy and 10 (16.13%) underwent lobectomy + segmentectomy.

**RESULTS:**

It was found that the rate of being asymptomatic was significantly higher in patients undergoing complete resection compared to those undergoing incomplete resection. Spirometric respiratory function tests were performed to assess the relationship between morphological type and the severity of disease. All parameters of respiratory function were worse in the saccular type and FEV_1_/FVC showed a worse obstructive deterioration in the saccular type compared to the tubular type.

**CONCLUSION:**

The success rate of the procedure increases with complete resection of the involved region. The morphological type is more important than the number and extension of the involved segments in showing the disease severity.

Bronchiectasis is the abnormal and persistent dilatation of the smaller bronchi and mainly the segmental or sub-segmental bronchi due to destruction of elastic tissues and muscles of the bronchial wall. Although the incidence has declined over the past years in societies with high socioeconomic status, bronchiectasis is still an important health problem in our country. Bacterial and viral infections are still ranked first in the etiology of bronchiectasis in developing countries, while immune deficiency syndromes (IgG, IgA deficiency and leukocyte dysfunctions), metabolic defects (cystic fibrosis, alpha-1 antitrypsin deficiency) and ultrastructural defects (primary cilliary dyskinesia, Young syndrome) are dominant in developed countries.[1]

In spite of medical treatment including physiotherapy, postural drainage and antibiotic treatment, the mortality rate for bronchiectasis has been reported to be 19-31%.[2]

Cases with long-term treatment lead a life without comfort and with high risk due to life-threatening complications. It is important to perform surgical treatment when indicated to save patients from these risks and to minimize the surgical risks in delayed cases.

The aim of this study was to review and present our cases that had undergone surgery for bronchiectasis in the past 12 years, the early and late term postoperative outcomes, our experience in bronchiectasis surgery, and the effect of morphological type on the prognosis.

## Methods

### Patients

Medical records of 62 cases undergoing surgical resection for bronchiectasis, from January 1995 to April 2009 in our clinic were assessed. Of our cases, 26 (42%) were outpatients. The remaining 36 cases (58%) were those referred from the Clinics of Pulmonary Diseases and Pediatrics. The demographic details, symptoms, surgical indications and procedures, rates of mortality and morbidity, and the functional status were assessed. Detailed medical history, physical examination, complete blood count and biochemical tests were reviewed. The mean age was 40.2±7 years and the age range was 9-71 years. Of the cases, 37 (59.68%) were female and 25 (40.32%) were male.

To assess the factors that played a role in the etiology, measles, pertussis and foreign body aspiration were questioned in the medical history. Family history, chest X-rays, PPD and gastric fluid culture were assessed for tuberculosis. Sweat test was evaluated for cystic fibrosis. For the diagnosis for humoral immune deficiency syndrome, IgG, IgA and IgM levels were measured with radial immune diffusion and the diagnosis was confirmed by lymph node biopsy. The criteria for the diagnosis of reactive airway disease were recurrent expiratory dyspnea attacks, history of familial atopy, positive prick test, elevated IgE level and obstructive type deterioration in PFT.

Of our cases, 70.96% were referred for surgical treatment due to inadequate conservative treatment. The disease was on the left in 33 (53.23%) cases, on the right in 26 (41.93%), and bilateral in three (4.84%) cases. A total of 66 operations were carried in 62 patients. The most common surgical procedure was [27 cases (43.54%)] lobectomy. Forty one (66.12%) patients underwent pneumonectomy, lobectomy and complete resection, including bilobectomy. Twenty-one (33.87%) cases underwent incomplete resection, of whom 11 (17.74%) underwent segmentectomy and 10 (16.13%) underwent lobectomy + segmentectomy.

Forty-four patients (70.96%) were referred to the surgery department after long-term medical treatment by the pulmonary disease specialists and pediatricians due to inadequate conservative treatment. These patients were the ones with persistent sputum production and recurrent infection attacks in spite of at least a 12-month treatment period. The other indications for surgery were: hemoptysis (n=7, 11.29%), destroyed lung (n=9, 14.51%) and bronchial obstruction due to the tumor (n=2, 3.22%).

The disease was bilateral in three cases (4.84%) and unilateral in 59 (96.16%) patients. The lesions were on the right side in 26 (41.93%) patients and on the left side in 33 (53.23%) patients. The mean number of involved segments was 4.2 (range: 1-11). There was ≤4 segment involvement in 39 (62.90%) cases. The most commonly involved locations were the inferior lobes (32.25%) [Table 1].

**Table 1:** Lung zones developing bronchiectasis

	Right	Left	Total (%)
Upper lobe	10	7	17 (27.41)
Middle lobe/lingula	8	3	11 (17.74)
Inferior lobe	9	11	20 (32.25)
Multilobar	4	9	13 (20.96)
Upper lobe anterior segment	1	2	3 (4.84)
Inferior lobe medial segment	2		2 (3.22)
Antero-medial basal segment	3	3	6 (9.67)
Middle lobe lateral segment	1		1 (1.61)
Inferior lobe posterio-basal segment	3	2	5 (8.06)

### Operation techniques

The period of time from the onset of symptoms to the operation showed variation between one and 30 years (mean: 21 months). All the patients underwent preoperative respiratory physiotherapy to optimize the respiration status. They were preoperatively cleared from secretions by therapeutic bronchoscopy. Using the results of the microbiological analysis of the bronchial aspiration, patients in whome it was indicated, received specific antibiotic treatment for two weeks. The physiotherapy program was continued until the daily sputum was ≤50 ml.

All the patients gave preoperative written consents. In the pre-anesthesia period, epidural catheter (Perifix^®^, B. Braun, Melsungen, Germany, Tuohy injector: 16 G, 100 mm) was placed at the level of thoracic 5-7, which was convenient for the surgical excision, using saline solution with the method of resistance loss while the patient was sitting or in the lateral decubitus position. A test dose of 3 ml 0.125% bupivacaine with 1/200.000 adrenalin was given via the epidural catheter and no problem was encountered. The first dose of 10 ml 0.125% bupivacaine + 2 μg/ml fentanyl were given postoperatively via the epidural catheter after the patient was awakened. Four ml/hour of the mixture of 0.125% bupivacaine and 2 μg/ml fentanyl was infused afterwards via the epidural catheter. The “Visual Analog Scale” (VAS) was used to assess the patients’ pain status. VAS scores were assessed just before the administration of analgesic methods, at every hour in the first 24 hours, and every three hours in the following days. A VAS scores at rest of ≥4 was accepted as insufficient analgesia and these patients received a bolus of 6 ml infusion solution. The infusion was reduced to 3 mg/kg/hour when bradycardia, hypotension, decrease in the number of respiration or deep sedation occurred.

The patients were intubated with double lumen endotracheal tube and were connected to controlled ventilation so that the tidal volume was 10 ml/kg and the frequency was 10 with 2% sevoflurane in 50/50% O_2_/air at inspiration.

Posterolateral thoracotomy was performed at the fifth intercostal space with patients at the lateral decubitus position. The type of resection was chosen according to the involved zone and cardiopulmonary reserve. Complete resection was defined as the anatomical resection of all affected segments. Lobectomy was performed if the disease was limited to one lobe and pneumonectomy was performed when one lung was involved. Segmentectomy was preferred when the PFTs were impaired. The aim was to resect as little segment as possible without compromising the aim of total cure of the disease.

Adhesions were released during the operation using electrocautery. All resections were performed using the standard techniques. Hilar vascular structures were isolated and ligated one by one. The ligation of large vessels was supported by 4/0 Proline suture material. Bronchi were closed up using 2/0 non-absorbable sutures. All resection specimens were examined histopathologically to confirm the diagnosis.

The majority of patients were extubated in the operation room. All the patients were followed up for at least 48 hours in the thoracic ICU.

Respiratory physiotherapy was implemented by a physiotherapy specialist as of the postoperative first day till the patients’ discharge from the hospital and was continued at home using the specialist’s instructions. The thoracic drain was extracted on the 2^nd^or 3^rd^postoperative day after the drainage, and/or when the air leakage was ceased. Although there was no reactivation of infection in the postoperative period, patients with the diagnosis of pulmonary tuberculosis received specific antibiotic prophylaxis for a minimum of three months.

### Definitions and statistical analysis

Cases with complete and incomplete resection were compared using the Z test for the improvement in clinical symptoms in the postoperative period and for the complete healing of the lesions.

The descriptive statistics median, mean, standard deviation, minimum and maximum values were used in the comparison of the features of tubular and saccular bronchiectasis.

The Mann-Whitney U test was performed to assess the difference between the groups. The Spearman correlation coefficient was also used to study the relationship between these features. The level of significance was set as 0.05 and calculations were implemented using the SPSS statistical pocket program.

## Results

The patients’ complaints on admission were, in decreasing order of frequency, productive cough (77.41%), recurrent pulmonary infections (66.1%), dyspnea (54.83%), sputum (43.55%), and hemoptysis (11.30%). Although 10 (16.13%) subjects stated that they were asymptomatic on admission, it was learnt from their medical histories that they had received intermittent medical treatment due to recurrent lower respiratory tract infection and 3 (30%) of them had to be hospitalized [Table 2].

**Table 2:** Patients’ symptoms

Symptoms		Sputum complaints		Characteristics of hemoptysis	
Productive cough	48 (77.41)	No sputum	35 (56.45)	No hemoptysis	55 (88.70)
Recurrent lower respiratory tract infection	41 (66.13)	Little amount of sputum (<100ml)	9 (14.52)	Pure blood	4 (6.46)
Dyspnea	34 (54.83)	Large amount of sputum (>100ml)	18 (29.03)	Mixed with sputum	3 (4.84)
Sputum with bad odor	27 (43.54)				
Fatigue	17 (27.41)				
Chest pain	7 (11.30)				
Hemoptysis	7 (11.30)				
Asymptomatic (during the application)	10 (16.13)					

Figures in parentheses are in percentage

The mean duration of the chronic symptoms was 5±2 years (1-24 years). Patients received medical treatment for a mean of 30±9 months (4 months - 21 years) before admission to our clinic.

All patients displayed pathological auscultation findings on the physical examination; approximately one third had dyspnea, 14.51% had clubbing, and 8.06% had chest deformity and barrel-shaped chest deformation as findings of long-term pulmonary pathology [Table 3].

**Table 3:** Findings of physical examination and accompanying diseases

Findings of physical examination		Accompanying diseases	
Ralles	62 (100)	COPD	11 (17.74)
Dullness and slight dullness	15 (24.19)	Sinusitis	9 (14.52)
Dyspnea	20 (32.25)	Heart failure	4 (6.46)
Hepato/ Splenomegaly	9 (14.52)	Renal disease	2 (3.22)
Tuber sufl	13 (20.96)	Hepatitis	4 (6.46)
Clubbing	9 (14.52)	Peptic ulcus	3 (4.84)
Otitis	4 (6.46)	Hypertension	8 (12.90)
Chest deformity and barrel-shaped chest	5 (8.06)	Malignancy	2 (3.22)
Cyanosis	7 (11.30)	Rheumatoid arthritis	2 (3.22)
		Interstitial lung disease	2 (3.22)

Figures in parentheses are in percentage

Of the cases, 27 (43.54%) had accompanying disease; 11 patients (17.74%) had COPD and 9 patients (14.52%) had sinusitis [Table 3].

The mean hemoglobin value was 12.42±1.95 g/dl; 16 patients (25.80%) had a hemoglobin value of <11g/dl. The mean leukocyte count was 3201.56±3760.04. Twenty seven patients (43.54%) had a leukocyte count of >10.000/mm3. The mean ESR was 24.23±28.10.

All the patients underwent pulmonary function test (PFT), except for three cases that were of young age (4.84%) with no compatibility for the test. Of these, 49% displayed normal PFT results, whereas 51% showed a mixed, obstructive respiratory pattern. The patients with low PFT results underwent quantitative pulmonary ventilation and perfusion scintigraphy.

The postero-anterior chest X-rays of all the patients revealed lesions of bronchiectasis. The diagnosis of bronchiectasis was supported with thoracic CT in 17 patients (27.41%) and with high resolution CT (HRCT) in 41 patients (66.13%). The lesions were assessed for morphology, localization and extensiveness and the number of lobes with bronchiectasis were counted. Using thoracic CT, morphological classification of 58 patients (93.54%) was made, revealing 65.52% to be tubular and 34.48% to be saccular. Before the routine use of thoracic tomography in our hospital, 4 patients (6.46%) had undergone diagnostic bronchography.

All patients suspected of endobronchial pathology underwent fiberoptic bronchoscopy (FOB).

The most common predisposing factor for bronchiectasis in our patients was chronic respiratory infections (29.03%) [Table 4]. Tuberculosis was in second place. It was found that in two patients (3.22%), bronchiectasis was caused by lung tumor. Biopsy with FOB from endobronchial lesions revealed one adenoma (located in the right upper lobe bronchi) and one pseudoinflammatory tumor (located in the right intermediary lobe bronchi). In one patient with bronchiectasis developing due to foreign body (Foxtails), the foreign body could be found at the end of the histopathological examination of the resected material.

**Table 4:** Etiology of bronchiectasis

Chronic lung infection	18 (29.03)
Pulmonary tuberculosis	11 (17.74)
Pulmonary sequestration	4 (6.45)
Obstruction due to foreign body	1 (1.61)
Immunodeficiency (IgG, IgA)	1 (1.61)
Cystic fibrosis	2 (3.22)
Lung tumor	2 (3.22)
Childhood infections	7 (11.30)
Measles pneumonia	4
Pertussis	3
Recurrent pneumonia	9 (14.52)
Reactive airway disease	2 (3.22)
Gastroesophageal reflux	1 (1.61)
Unknown etiology	4 (6.45)

Figures in parentheses are in percentage

There was no bacterial growth in the sputum culture in 18 (29.03%) patients. Mixed growth was found in the others, with gram-negative organisms in the majority. There was growth of *Escherichia coli* and *Klebsiella* in 13 cases (20.97%), *Haemophilus influenzae* in 11 cases (17.74%), Pseudomonas in seven cases (11.29%), and *Streptococcus pneumoniae* in eight cases (12.90%). The sputum was acid-fast bacillus-positive preoperatively in five cases (8.06%) and these patients were given antituberculosis treatment. They were operated after the sputum culture turned negative.

A total of 66 operations were performed on 62 cases. One case underwent complementary right pneumonectomy 10 months after right lower lobectomy. Three cases with bilateral involvement underwent right lower lobectomy after left lower lobectomy. The most common procedure [27 (43.54%)] was lobectomy. Segmentectomy was preferred in those with limited pulmonary function parameters. Furthermore, decortication was performed in six cases and apical bulla ligation and pleurectomy were performed in two cases for the accompanying lesions [Table 5]. Forty-one cases (66.12%) with pneumonectomy, lobectomy and bilobectomy were accepted as complete resection (1 case of lobectomy + complementary pneumonectomy, 3 cases of bilateral lobectomy, 45 complete resections), and 21 cases (33.87%) with lobectomy + segmentectomy and segmentectomy were accepted as incomplete resection. The patients with incomplete resection were the ones with impaired PFTs that could not undergo lobectomy.

**Table 5:** Surgical procedures in 62 patients with bronchiectasis

Procedure	No. of patients
Pneumonectomy	13 (20.96)
Left	9
Right	4
Lobectomy	27 (43.54)
Right upper lobe	4
Left upper lobe	7
Right lower lobe	7
Left lower lobe	9
Bilobectomy	5 (8.06)
Right upper lobe + middle lobe	4
Right lower lobe + middle lobe	1
Lobectomy + segmentectomy	10 (16.13)
Right middle lobe + upper lobe anterior segment	1
Left lower lobe + Lingula	2
Right lower lobe + lower lobe medial segment	1
Right middle lobe + lower lobe medial segment	1
Right upper lobe + lower lobe posteriobasal segment	1
Right upper lobe + middle lobe lateral segment	1
Right middle lobe + anteriomedial basal segment	3
Segmentectomy	11 (17.74)
Left upper lobe anterior	2
Lingula	1
Right posterobasal segment	2
Left posterobasal segment	3
Left anteromedial basal segment	3

Figures in parentheses are in percentage

The pathology results were chronic infection and bronchiectasis, which were compatible with the preoperative diagnoses. The histopathological examination confirmed the endoscopic diagnosis in one patient with bronchial adenoma and in one patient with pseudotumor. In one patient, foxtail weed was detected in the tissue in the pathological specimen.

The number of involved segments, the time of hospital admission the PFT results and the morphological classification of 58 (93.54%) cases that had been assessed by thoracic CT (65.52% tubular, 34.48% saccular) have been presented in Tables 6 and 7.

**Table 6:** Analysis of tubular and saccular type according to studied parameters

	Tubular					Saccular					*P*
	Median	Mean	St. dev.	Max.	Min.	Median	Mean	St. dev.	Max.	Min.	
Age	29.00	33.64	15.42	71.00	11.00	14.00	18.85	9.38	45.00	9.00	0.001
Time of admission	6.00	6.28	4.08	18.00	1.00	13.00	13.40	5.52	24.00	4.00	0.001
The number of involved segment	4.00	5.23	2.29	11.00	2.00	4.00	5.10	2.22	9.00	2.00	0.862
FVC	2.11	2.14	.36	2.85	1.56	1.70	1.80	.53	2.61	1.11	0.012
FEV_1_	2.28	2.28	.26	2.77	1.61	1.43	1.42	.08	1.61	1.26	0.000
VC	2.26	2.28	.23	2.71	1.78	2.13	2.14	.19	2.56	1.78	0.030
FEV_1_.FEVC	83.00	80.77	8.22	96.00	61.00	64.50	65.75	6.21	79.00	57.00	0.001

**Table 7:** The Spearman correlation coefficient of features for the tubular and saccular groups

	Age	Time of admission	Involved segment	FVC	FEV_1_	VC	FEV_1_.FEVC
Tubular group							
Age	1						
Time of admission	.271	1					
Involved segment	-.017	.008	1				
FVC	-.037	-.217	.134	1			
FEV_1_	-.356*	.060	.223	.012	1		
VC	-.040	.072	-.263	-.571**	.025	1	
FEV_1_.FEVC	-.036	-.227	-.391*	-.108	-.292	-.002	1
Saccular group							
Age	1						
Time of admission	-.613**	1					
Involved segment	-.047	.102	1				
FVC	.023	-.271	.084	1			
FEV_1_	.049	.014	.224	.147	1		
VC	-.173	.099	.013	.352	.250	1	
FEV_1_.FEVC	.346	-.222	-.135	.173	.058	.384	1

* :*P*<0.05

** :*P*<0.01

The time of hospital admission was significantly shorter in cases with tubular morphology than in cases with saccular type (*P*=0.001). As the time for admission was extended and as the patient age increased (*P*=0.001), the possibility of having saccular type also increased. There was no significant relationship between the morphological type and the number of involved segments (*P*=0.862). Spirometric PFT was performed to assess the relationship between the morphological type and the severity of the disease. All PFT parameters were worse in the saccular type [(FVC, *P*=0.012), (VC, *P*=0.03), (FEV_1_, *P*=0.0001)]. FEV_1_/FVC values showed a worse obstructive deterioration as an important finding in the saccular type than in the tubular type (*P*=0.001).

There was no operative or postoperative mortality. The mean duration of hospital stay was 9.4 days (range: 5-24 days). There were complications in 12 cases and the morbidity rate was 19.35%. Bronchoscopic aspiration was needed in four cases (6.45%) due to sputum retention. There was persistent air leakage for more than 10 days in two cases (3.22%), empyema in two cases (3.22%) and bronchopleural fistula in two patients (3.22%). Two cases with empyema recovered with intercostal drainage and long-term antibiotic treatment. Both the patients with bronchopleural fistula had associated infection; thoracostomy with an Eloesser flap was performed in the early postoperative period. Bronchopleural fistula closure with a pectoralis major muscle flap and window closure were carried out in both patients. Re-thoracotomy was needed in one patient (1.61%) due to postoperative hemorrhage. Severe supraventricular arrhythmia occurred in one patient (1.61%) and was managed by pharmacological intervention [Table 8]. No difference was found in terms of postoperative complication rates according to morphologic types of bronchiectasis; howeer, the most severe postoperative complication, broncho-pleural fistula, developed in 2 (3.22%) cases with saccular bronchiectasis.

**Table 8:** Complications of the operation

Complication	No. of patients (%)
Sputum retention and atelectasis	4 (6.45)
Persistent air leak for more than 10 days	2 (3.22)
Empyema	2 (3.22)
Bronchopleural fistula	2 (3.22)
Postoperative hemorrhage	1 (1.61)
Severe supraventricular arrhythmias	1 (1.61)

Sixty patients were followed-up for a mean period of 4.5 years (range: 1-10 years). Two patients (3.22%) who underwent lobectomy who lived in other cities could not be followed-up.

The patients were assessed clinically during the follow-up. It was found that 27 patients (45%) were asymptomatic, 25 patients (41.67%) showed improvement in symptoms compared to the pre-operative period. There was no improvement in the symptoms in eight patients (13.13%).

Being asymptomatic was significantly higher in the group undergoing complete resection than those undergoing incomplete resection (*P*<0.046). There was no significant difference in the improvement of symptoms compared to the preoperative period in these two groups (*P*=0.678). The rate of patients with incomplete resection was significantly higher within the patients with no improvement (*P*<0.004) [Table 9]. The clinical status of patients according to the resection type has been presented in Table 10.

**Table 9:** Resection type and clinical status

	Patients with complete resection (n=39)	Patients with incomplete resection (n=21)	
Asymptomatic	21	6	Z = 1.99 *P* < 0.046
Clinical improvement	17	8	Z = 0.410 *P* = 0.678
No clinical improvement	1	7	Z = 2.900 *P* < 0.004

**Table 10:** Postoperative symptoms according to the type of resection

Type of resection	Asymptomatic + clinical improvement	Symptoms worse/same
Pneumonectomy	13	
Lobectomy	20	1
Bilobectomy	5	
Lobectomy + segmentectomy	7	3
Segmentectomy	7	4

## Discussion

While the prevalence of bronchiectasis is decreasing in industrialized countries due to regular vaccination of children and effective treatment of pulmonary infections with specific antibiotics, it still seems a chronic disease and a surgical problem in developing countries.[3]

The prevalence of bronchiectasis has decreased, especially in developed countries, due to preventive measures, and adequate treatment of childhood infections and tuberculosis. It is rare in developed countries, except in cases with cystic fibrosis (CF), and the causes except CF, are grouped in one main group named “non-CF bronchiectasis”.[4] Sweat chloride test, repeated sweat chloride tests in suspected cases, and mutation analysis were performed for CF, and we found the rate of secondary bronchiectasis due to CF in our study to be quite low (3.22%). However, a higher rate of CF (17%) in our country suggests that there may be regional variations in the etiology.[5]

Widespread and effective immunization programs have led to a significant reduction in the prevalence and respiratory complications of pertussis and measles.[6] In our country, in spite of the relatively successful vaccination campaigns, measles, pertussis and tuberculosis are still important as infectious factors with the potential of developing bronchiectasis. The rate of bronchiectasis due to these childhood infections in our study was 11.30% [Table 4].

Recurrent infections with inadequate treatment of pulmonary infections were the most common (29.03%) cause of bronchiectasis in our series, similar to the other series.[7–9]

Tuberculosis is still one of the most important causes of bronchiectasis in developing countries. Bronchiectasis usually develops in the same pulmonary region 1-3 months after the onset of the infection.[5,10] This complication occurs in approximately 11% of the patients with tuberculosis. History of tuberculosis was detected in 84.2% of the 120 patients who had undergone monitoring for bronchiectasis in a series from the northeastern part of Brazil.[11] The incidence of tuberculosis is still high in Turkey and it has an important role in the etiology of bronchiectasis.[5,7,12]

In our study, tuberculosis was in second rank in etiological factors. This shows the importance of tuberculosis in the etiology of bronchiectasis.

Although bronchiectasis is defined as a childhood disease,[13,14] the mean age of our patients was higher (40.2±7). The reasons for the older age may be listed as follows: late admission to a physician, previous empiric treatments in patients with no radiological investigation, resistant microorganisms due to irrational antibiotic use, which is very common in Turkey (inappropriate and/or inadequate antibiotic use, antibiotic use without physician prescription), smoking prevalence, and deterioration in the general health conditions. Due to these factors, our patients who had no genetic or immunological defects became prone to the recurrent chronic infections.

The main complaints of patients with bronchiectasis were chronic cough, purulent sputum, hemoptysis, dyspnea, wheezing, fatigue, fever and failure to thrive. Recurrent and persistent pulmonary infections cause the prominence of these symptoms. The diagnosis may be incidental in some cases with no symptoms when chest X-ray or CT is performed for other reasons.[15]

Copious sputum may not be a prominent symptom, especially in cases with upper lobe localization; hence, these are referred to as “dry bronchiectasis”. The bronchiectasis is classified as mild if the daily sputum amount is less than 10 ml, as moderate if it is 10-150 ml, and as severe if the amount is >150 ml.[16] Our cases were assessed in three groups: no sputum, <100 ml, and >100 ml. There was no history of sputum production in 56.45% of cases. Of these, only five patients had a history of tuberculosis. The high rate of patients (56.45%) without sputum was considered to be related to antibiotic use in the early period.

Hemoptysis is a common symptom in bronchiectasis and is more common in cases with dry bronchiectasis.[1,5,14] It usually originates from the anastomosis between bronchial arteries and pulmonary vessels or aneurysms of the bronchial artery. It is seen in 50% of cases with bronchiectasis.[1,5,14,17] Our rate for hemoptysis was 11.30%.

Nicotra *et al*.[17] reported dyspnea in 70% of cases with bronchiectasis. Our rate for dyspnea was 54.83%.

Although chest X-rays support the diagnosis in patients who are suspected to have bronchiectasis based on clinical findings, the definitive diagnosis is made by thoracic CT or bronchography.[1,17] We believe that thoracic CT is satisfactory in addition to PA and lateral chest X-rays for the assessment of bronchiectasis. This opinion has many advocates[18,19] because pathological data and thoracic CT findings are consistent in almost all aspects. Thus, CT diagnosis in all our cases was consistent with the postoperative pathological diagnosis (sensitivity: 100%).

We found that the prevalence of the saccular type with 36% in our cases was more marked than the tubular type in older cases and in cases with longer periods having passed till admission. Irrespective of gender, as the time for admission prolonged, the saccular type was dominant compared to the tubular type in the morphology, despite the fact that they had an equal number of bronchiectatic areas. This condition indicated that the morphological type is as important as the number and size of the involved segments in terms of predicting the severity of the disease. Thus, the saccular type can be accepted as an indicator of progression of the underlying disease and an important prognostic factor. Therefore, thoracic CT can be a useful index, not only for the definitive diagnosis of bronchiectasis, but also for the determination of the severity of the disease by determining the morphology. This finding supports the opinion by Lynch *et al*.:[20] “defining the morphological type of bronchiectasis by thoracic CT can be an index indicating the functional severity of the disease”.

The left lung is most commonly involved in bronchiectasis. The possible causes are: the left main bronchi is narrower than the right; peribronchial area in the left lung is under pressure by the aortic arch and the lymph nodes; there is a higher possibility for the left main bronchi to be under pressure compared to right, as it is longer. Although there are various rates for the involvement of the left lung in different publications, the rate has been reported as 55%-80%.[2,12] The rate for the bilateral localized involvement has been reported as 3.6%-19%. We found left lung involvement as 53.22% and bilateral involvement as 4.84%. It was seen that bronchiectasis most commonly involved the lower lobes in our cases, (32.25%) [Table 1].

In spite of the developments in medical treatment, some patients still need surgery.[2,21] In more than half of surgical applications as seen in our study, the reasons were: inadequate medical treatment followed by hemoptysis, lung abscess, and mass lesion.[2,22] The low morbidity and mortality rates of of the surgical outcomes and their success in preventing the progression of the disease show that surgical treatment is a good alternative to medical treatment.

Surgical resection of bronchiectasis has played an important role in the treatment of disease in developing countries since the 1950s.[23,24] Among thoracic surgeons, the opinion that the indication for operation being limited to localized bronchiectasis and surgical treatment being contraindicated for bilateral bronchiectasis has been accepted, especially at the end of 1970s.[21,22,25]

After the age of 17, patients with bronchiectasis become patients of Pulmonary Medicine instead of Pediatrics. After several years, they are referred to the surgery department. The rate of total cure is 75-100% in childhood bronchiectasis in the group undergoing surgical treatment. On the other hand, it is approximately 40% in the group with medical treatment.[26] Of our cases, 33.87% (9-17 years of age, 21 cases) were children. The indication for surgery for the majority of this group was recurrent infections. Childhood bronchiectasis was most common in the 11-17 years age group in our series, and this was consistent with the literature.[26] All cases except for one with bilateral disease had total cure.

The philosophy of the current surgical treatment is to protect as much healthy tissue as possible and to completely remove diseased tissues.[27,28]

In one study, fibrosis due to destruction of the lung parenchyme (destroyed lung) was found to be secondary to tuberculosis in 19% of cases.[28] The rate of pneumectomy due to tuberculosis was reported as 28.5% in another study.[29] The rate of subjects who underwent pneumectomy because of tuberculosus bronchiectasis is 72.72% (8/11) in our series. The population of our region comprises individuals in low socio-economic status and low educational status. Health services are inadequate in our region. Due to all these reasons, it is not always possible to diagnose tuberculosis and similar diseases on time and to treat them effectively. Moreover, effective monitorization is lacking about whether the patients use their medications appropriately or not and about the progression of the disaese, even if a diagnosis is made.

This condition leads to an increase in the rate of destructive lung development due to the current disease and increases the rate of pneumectomies, as in our series (20.96%) [Tables 4 and 5]. Multilobar involvement was detected on the radiological imaging of our pneumectomized patients, the mean age of whom (56.7±8.2) was higher than all cases (40.2±7). The destroyed lung was the indication for pneumectomy in eight of them (61.53%). The remaining four (38.47%) were operated on for hemoptysis episodes accompanied by a productive cough, abundant sputum, respiratory distress, and fatigue. One subject underwent right lower lobectomy first, because of lower lobe destruction along with cylindrical and varicose bronchiectasis in the right upper and medial lobes; however, pneumectomy was performed at the 10 month due to lack of improvement in symptoms in the postoperative period.

The rate of postoperative complication in cases with bronchiectasis is 15-25% and the surgical mortality is 2%.[2,21,29,30] The surgical morbidity in our study was 19.35% and we had no mortalities. The most common resection type in our series was lobectomy (43.54%) and the rate for segmentectomy was only 17.74%. These results show that it is possible to perform successful complete resection with low morbidity and mortality in bronchiectasis. It was found that cases that had poor outcomes in spite of surgical treatment were those with extensive and complicated bronchiectasis.[21] The success rate is reduced in spite of surgical treatment especially in cases with the following features: detected pseudomonas, permanent obstructive pathology, resected perfused segments and bilateral diffusion.[2] This finding suggests that the success rate may be higher if surgical intervention is performed before the development of complications. Therefore, surgical resection may be more limited.[22] Ashour *et al*.[2] emphasized that giving priority to the resection of non-perfused areas and protecting the perfused areas, especially in cases with bilateral involvement and assessing the patient preoperatively for this may play a significant role in the surgical success. In fact, the type with functional loss is the saccular/cystic type. The resection of such an area does not cause a loss of the patient’s pulmonary functions.

Ashour[2] stated that pulmonary perfusion was retained in the area of cylindrical changes and that, therefore, this type was not a primary indication for surgical management. However, recurrent infections resistant to treatment is the indication for surgical treatment in cylindirical bronchiectasis. Moreover, many studies have indicated that postoperative prognosis is better in cylindirical bronchiectasis types operated on compared to the other types, because of resistant infections to long term medical treatment.[8,10]

Complete resection was possible in the majority (66.12%) of our cases. Surgical management is not contraindicated in selected patients with bilateral bronchiectasis.[27,28] In our series, three patients (4.84%) had bilateral bronchiectasis and they underwent left lower lobectomy, followed by right lower lobectomy.

Of the 60 cases with follow-up, 52 (86.66%) were asymptomatic and showed clinical improvement. Of these, 66.33% were the ones with complete resection. These results were consistent with those in the literature.[14,29]

In conclusion, surgical resection for bronchiectasis has acceptable morbidity and mortality rates for each age group. The success rate of the procedure increases with complete resection of the affected section. This study showed that the type with more peripheral and severe deterioration was the saccular type, regardless of gender and the extent of the segmental involvement. It is again the saccular type with significant reduction in the pulmonary functions parallel to older age, longer time before admission and inadequate medical treatment. Therefore, we think that the saccular type is an important prognostic factor. It has been concluded that correct timing of the surgical intervention when performed before the development of obstructive pathology and at younger age can enable reduction in the postoperative morbidity and mortality and can be more effective.

## References

[1] Swartz MN, Fishman AP, Elias JA, Grippi MA, Kaiser LK, Senior RM (1998). Bronchiectasis. Fishman’s Pulmonary disease and disorders.

[2] Ashour M, Al-Kattan KM, Jain SK, Al-Majed S, Al-Kassimi F (1996). Surgery for unilateral bronchiectasis: Results and prognostic factors. Tuber Lung Dis.

[3] Aghajanzadeh M, Sarshad A, Amani H, Alavy A (2006). Surgical management of bilateral bronchiectases: Results in 29 patients. Asian Cardiovasc Thorac Ann.

[4] Chang AB, Masel JP, Boyce NC, Wheaton G, Torzillo PJ (2003). Non-CF bronhiectasis: Clinical and HRCT evaluation. Pediatr Pulmonol.

[5] Karakoç GB, Yılmaz M, Altıntaş DU, Kendirli SG (2001). Bronchiectasis: Still a problem. Pediatr Pulmonol.

[6] Nikolaizik WH, Warner JO (1994). Etiology of chronic suppurative lung disease. Arch Dis Child.

[7] Eren S, Esme H, Avci A (2007). Risk factors affecting outcome and morbidity in the surgical management of bronchiectasis. J Thorac Cardiovasc Surg.

[8] Fujimoto T, Hillejan L, Stamatis G (2001). Current strategy for surgical management of bronchiectasis. Ann Thorac Surg.

[9] Prieto D, Bernardo J, Matos MJ, Eugenio L, Antunes M (2001). Surgery for bronchiectasis. Eur J Cardiothorac Surg.

[10] Haciibrahimoglu G, Fazlioglu M, Olcmen A, Gurses A, Bedirhan MA (2004). Surgical management of childhood bronchiectasis due to infectious disease. J Thorac Cardiovasc Surg.

[11] Bogossian M, Santoro IL, Jamnik S, Romaldini H (1998). Bronchiectasis: A study of 314 cases tuberculosis × non-tuberculosis. J Pneumol.

[12] Kutlay H, Cangir AK, Enon S, Sahin E, Akal M, Gungor A (2002). Surgical treatment in bronchiectasis: Analysis of 166 patients. Eur J Cardiothorac Surg.

[13] Balkanlı K, Genç O, Dakak M, Gürkok S, Gözübüyük A, Çaylak H (2003). Surgical management of bronchiectasis: Analysis and short-term results in 238 patients. Eur J Cardiothorac Surg.

[14] Stephen T, Thankachen R, Madhu AP, Neelakantan N, Shukla V, Korula RJ (2007). Surgical results in bronchiectasis: Analysis of 149 patients. Asian Cardiovasc Thorac Ann.

[15] Javidan-Nejad C, Bhalla S (2009). Bronchiectasis. Radiol Clin North Am.

[16] Ethan EE, Ouellette DR, Hollingsworth HM, Talavera F, Ouellette DR, Rice TD, Mosenifar Z “Bronchiectasis.” eMedicine. http://www.emedicine.com/med/topic246.htm.

[17] Nicotra MB, Rivera M, Dale AM, Sheherd R, Carter R (1995). Clinical, pathophysiologic, and microbiologic characterization of bronchiectasis in an aging cohent. Chest.

[18] Vernhet H, Bousquet C, Cover S, Giron J, Gruson C, Senac JP (1995). The use of high resolution X-ray computed tomography in the diagnosis of hypersensitivity pneumopathy to gold salts. Apropos of a case. Rev Mal Respir.

[19] Kang EY, Miller RR, Müller NL (1995). Bronchiectasis: Comparison of preoperative thin-section CT and pathologic findings in resected specimens. Radiology.

[20] Lynch DA, Newell J, Hale V, Dyer D, Corkeey K, Fox NL (1999). Correlation of CT findings with clinical evaluations in 261 patients with symptomatic bronchiectasis. AJR Am J Roentgenol.

[21] Etiene T, Spiliopoulos A, Megevand R (1993). Bronchiectasis: Indication and timing for surgery. Ann Chir.

[22] Agasthian T, Deschamps C, Trastek VF, Allen MS, Pairolero PC (1996). Surgical management of bronchiectasis. Ann Thorac Surg.

[23] Annest LS, Kratz JM, Crawford FA (1982). Current results of treatment of bronchiectasis. J Thorac Cardiovasc Surg.

[24] Miller JI, Shields TW, LoCicero J, Reed CE, Feins RH (2000). Bronchiectasis. General thoracic surgery.

[25] George SA, Leonardi HK, Overholt RH (1979). Bilateral pulmonary resection for bronchiectasis: A 40-year experience. Ann Thorac Surg.

[26] Pitney AC, Callahan CW, Ruess L (2001). Reversal of bronchiectasis caused by chronic aspiration in cri du chat syndrome. Arch Dis Child.

[27] Giovannetti R, Alifano M, Stefani A, Legras A, Grigoroiu M, Collet JY (2008). Surgical treatment of bronchiectasis: Early and long-term results. Interact Cardiovasc Thorac Surg.

[28] Halezeroglu S, Keles M, Uysal A, Celik M, Senol C, Haciibrahimoglu G (1997). Factors affecting postoperative morbidity and mortality in destroyed lung. Ann Thorac Surg.

[29] Dezfouli AB, Kakhki AD, Farzanegan R, Javaherzadeh M (2003). Results of lobectomy and pneumonectomy in pulmonary TB. Tanaffos.

[30] Gursoy S, Ozturk AA, Ucveti A, Erbay AE (2010). Surgical management of bronchiectasis: The indications and outcomes. Surg Today.

